# Validation of the Chinese version of the Amsterdam Preoperative Anxiety and Information Scale (APAIS)

**DOI:** 10.1186/s12955-020-01294-3

**Published:** 2020-03-11

**Authors:** Hao Wu, Xin Zhao, Shuaishuai Chu, Fangxia Xu, Jia Song, Zhengliang Ma, Xiaoping Gu

**Affiliations:** Department of Anesthesiology, Nanjing Drum Tower Hospital, The Affiliated Hospital of Nanjing University Medical School, 321 Zhong Shan Road, Nanjing, Jiangsu 210008 People’s Republic of China

**Keywords:** Preoperative anxiety, Questionnaires, Validation studies, Coping styles

## Abstract

**Background:**

Preoperative anxiety is an unpleasant state of tension that may impact patients’ post-operative pain and satisfaction. The level of preoperative anxiety should be routinely identified. The Amsterdam Preoperative Anxiety and Information Scale (APAIS) is a self-reported questionnaire that is used to quickly assess preoperative anxiety and information needs with good psychometric properties.

**Objectives:**

To validate the Chinese version of the Amsterdam Preoperative Anxiety and Information Scale (APAIS) and to explore coping strategies used by patients in dealing with surgery and anesthetic.

**Methods:**

The cross-cultural validation of APAIS involved the translation of a Chinese version of APAIS and an investigation of its psychometric properties and clinical applicability. Forward-back translation and a pilot study were performed to produce a Chinese adaptation of APAIS. The inpatients of the orthopedic, otolaryngology, and general surgery department scheduled for general anesthesia surgery were enrolled to complete psychometric testing. The reliability was assessed using Cronbach’s alpha. Exploratory factor analysis and confirmatory factor analysis were calculated to assess construct validity. The criteria validity was analyzed using the correlation between APAIS and State-trait anxiety inventory-state (STAI-S) and Visual analogue scale-anxiety (VAS-A). Coping styles were evaluated using the Medical Coping Modes Questionnaire (MCMQ) score that covered three domains: confrontation, avoidance, and resignation. The impact of different coping styles on patients’ anxiety was explored.

**Results:**

A total of 204 valid questionnaires were collected the day before surgery. Cronbach’s alpha coefficients were 0.862 for the anxiety scale and 0.830 for the information scale. Exploratory factor analysis with oblique rotation revealed two factors that explained 76.45% of the total variances. A confirmatory factor analysis showed a two-factor model with an adequate model fit (root mean square error of approximation: 0.073, goodness-of-fit: 0.966). The APAIS anxiety score significantly correlated with STAI-S (*r* = 0.717, *P* < 0.01) and VAS-A (*r* = 0.720, *P* < 0.01). For the three coping strategies, preoperative anxiety had a low correlation with confrontation (*r* = 0.33, *P* < 0.01) and resignation (*r* = 0.22, *P* < 0.05).

**Conclusions:**

The Chinese version of APAIS is a valid and reliable instrument for assessing preoperative anxiety. Use of this measurement tool for Chinese patients is feasible and shows promising results.

## Background

Preoperative anxiety is an unpleasant state of tension and a worldwide problem in the perioperative period. Excessive anxiety can lead to physiological and psychological responses including hypertension, arrhythmias, severer postoperative pain, depression that may impact patients’ prognosis and post-operative satisfaction [1, 2]. As proper psychological intervention can relieve anxiety and improve patient outcomes, patients with a high degree of anxiety should be routinely identified during the anesthesiologist’s preoperative interview [2, 3]. Applicable screening instruments that are suitable for preoperative interview settings should be brief and understandable, in addition to reliable and valid [4]. The Amsterdam Preoperative Anxiety and Information Scale (APAIS) is a self-reported questionnaire developed by Moerman in Dutch used to quickly assess preoperative anxiety with good clinical relevance. The scale consists of six items with a simple analysis format. It can be divided into two scales, including an anxiety scale (items 1, 2, 4, and 5; Cronbach’s α = 0.86) and a need-for-information scale (items 3 and 6; Cronbach’s α = 0.72). The APAIS anxiety score is highly correlated with State-trait anxiety inventory-state (STAI-S) [5]. APAIS has been translated into many languages, and the recent version included English, Japanese, French, German, Italian, and Spanish [6–11]. The cultural difference have been found in evaluation of anxiety. The level of social related anxiety in China was higher than England and Switzer⁃land, but showed no significant differences compared to Japan and Indonesia. The impact of cultural influences to preoperative anxiety were not clear [12, 13]. Patient’s psychological feeling and satisfaction have been paid more attention in developing countries compared with the past. The high levels of acceptance and clinical relativity make APAIS a standard tool to measure preoperative anxiety that can contribute to improving perioperative psychological feeling in Chinese patients. The primary aim of this study was to develop a Chinese version of APAIS and evaluate its psychometric properties. The correlations between APAIS and STAI-S, Visual analogue scale-anxiety (VAS-A) were evaluated to assess criteria validity. Exploratory factor analysis and confirmatory factor analysis were applied to assess construct validity.

Patients respond to stress in different ways. According to the coping style classified by APAIS, patients who need more information tend to be “blunters”, whereas “monitors” require less information support. Coping style is not only an explanatory variability in response to stress, but also an intervention between stress and emotional distress [14]. More specifically, in preoperative stage, “blunter” might benefit from more information and attention. However, extensive information is not useful for those patients with a “blunting” coping style and may even induce excessive anxiety [5]. An important coping task is to regulate stress-induced negative emotions, especially distress [14, 15]. The secondary aim of this research is to explore different coping strategies used by patients in dealing with surgery and anesthetic, and the effect of different coping styles on preoperative anxiety.

## Method

### Study population and ethical issues

This study was approved by the Ethics Committee of the Affiliated Drum Tower Hospital of the Medical Department of Nanjing University (2015–011-01) on March 2, 2015, and was conducted in accordance with the ethical principles of Helsinki Declaration. All patients were told of their related rights and signed informed consent. Authorization for the Chinese version of APAIS was approved by the original author N. Moerman. Inpatients at the orthopedic, otolaryngologic, and general surgery departments scheduled for general anesthesia surgery between September 2015 were asked to participate in the study. The inclusion criteria were (1) patients undergoing general anesthesia for elective surgery; (2) age > 18 years; (3) physical status of American Society of Anesthesiologists (ASA) I-III; (4) patients were able to read and understand the questionnaire; and (5) they signed informed consent. Patients with psychiatric illnesses, poor medical conditions (heart failure, respiratory failure, shock and multi-organ failure), inability to answer the questionnaire, and who did not consent were excluded.

### Development of Chinese version of APAIS

We translated APAIS according to the rules of the European Organization for the Research and Treatment of Cancer translation procedure [5] and the guidelines of adaptation and validation of instruments in cross-cultural health care research [16], which include three main steps: forward translations, blind back-translation, and pilot testing of the preliminary version. A bilingual doctor of anesthesiology and a bilingual graduate majored in English were required to translate APAIS into Chinese. After ambiguities and discrepancies of two translated versions were compared, the initial translated version was generated. Blind back-translation of the initial translated version of were accomplished by two other bilingual independent translators who majored in anesthesiology and English, respectively. The words, sentence structure, semantics of back-translation were discussed and the preliminary version of APAIS was formed. After translation, the preliminary version was applied to the pilot studies; 30 patients (13 males and 17 females) were involved, with an average age of 43. They completed the questionnaire within 2 minutes and reported no misunderstanding, which ensured that the acceptability for each dimension was good.

### Psychometric testing of the Chinese APAIS

We tested the psychometric properties of the Chinese version APAIS. Data from the APAIS item scores were randomly divided into two groups. Exploratory factor analysis was used in one group to explore the structural relationship between the six items and a confirmatory factor analysis was applied to the other group to verify the structure model. To assess the criteria validity, we analyzed the correlation between APAIS and STAI-S, and VAS-A. In the preoperative stage, STAI-S is a criteria instrument for measuring the current situation-related anxiety and a widely used instrument that have been translated and validated into Chinese. The VAS-A has characteristics of simple form and well-understood that showed good reliability and validity. The reliability of APAIS anxiety sub-scale and APAIS information sub-scale were assessed using Cronbach’s α coefficient. Student’s t-test was used to compare the mean scale scores.

### Questionnaires

#### APAIS

APAIS is a 6-item questionnaire that can be subdivided into 2 major components. In the anxiety scale, items 1 and 2 assess anesthesia-related anxiety, and items 4 and 5 evaluate surgery-related anxiety. In the need-for-information scale, items 3 and 6 assess the desire for information about anesthesia and surgery. All questions range from 1 (“not at all”) to 5 (“extremely”). The score of the total anxiety scale ranges from 4 to 20 and 2 to 10 for the information desire score [5].

#### STAI-S

STAI-S is a widely used instrument and the gold standard for assessing preoperative anxiety. It was translated and adapted into Chinese with good reliability and validity. It consists of 20 items for measuring the current situation-related anxiety, with scores ranging from 20 to 80 [17].

#### VAS-A

The VAS-A consists of a 100 mm horizontal line; the left edge of the line is marked as “calm,” while the other end shows “maximum anxiety.” The patients were asked to assess their own anxiety and mark it on the anxiety line [18].

#### MCMQ

The coping style was measured using the MCMQ, which is a 20-item questionnaire that rates on a 4-point Likert-type scale (“never” to “very little;” “all the time” to “very much”). The item scores are summed to produce confrontation, avoidance, and resignation levels, which range from 0 to 27, 0 to 21, and 0 to 15. The Chinese version of the MCMQ has evidence of satisfactory validity and reliability. In previous study, Cronbach’s α of an eight-item confrontation scale, a seven-item avoidance scale, and a five-item acceptance-resignation scale were 0.70, 0.66, and 0.67, respectively [19].

### Data collection

Two types questionnaires were prepared, the first type of questionnaires containing APAIS, STAI-S, and VAS-A, and the second type of questionnaires containing APAIS, STAI-S, VAS-A and MCMQ. Two kinds of questionnaires with equal number of 120 were randomly to provide to eligible participants the day before surgery. Two hundred twenty-four patients agreed to participate and 204 patients completed the questionnaires before surgery. The distribution and collection of questionnaires were done by one anesthetist. The patients’ demographic information was collected, included age, sex, educational background, previous surgeries, and underlying illnesses. The surgery type was divided into minor, intermediate, and major surgery as in the original version.

## Results

### Study population and item scores

The Chinese version of APAIS is shown in Table 1. A total of 204 valid questionnaires were collected the day before surgery; 101 respondents completed the anxiety MCMQ and there were no significant differences between the demographic data of two types questionnaires. The participants were 53.9% male and 46.1% female, and the mean age of the inpatients was 47.4 ± 14.8 years. Overall, 44.1% of the participants had a history of previous surgeries. The respondents’ demographic characteristics and mean APAIS scores are shown in Table 2. The mean of total anxiety score was 8.2 ± 3.6, the anesthesia-related anxiety score was 3.7 ± 1.8, the surgery-related anxiety score was 4.5 ± 2.2, and the information desire component was 5.4 ± 2.6.

**Table 1:** List of the 6 APAIS items

Original items	Chinese items
I am worried about the anesthetic.	我对麻醉感到担心
The anesthetic is on my mind continually.	我一直在想麻醉这件事
I would like to know as much as possible about the anesthetic.	我希望尽可能多地了解有关麻醉的事
I am worried about the procedure.	我对手术感到担心
The procedure is on my mind continually.	我一直在想手术这件事
I would like to know as much as possible about the procedure.	我希望尽可能多地了解有关手术的事

**Table 2:** Characteristics and APAIS score of the sample

		Total anxiety score	Information desire
Variable	n (%)	Mean (SD)	Mean (SD)
Sex			
Male, No. (%)	110	7.5 ± 3.3	5.6 ± 2.7
Female, No. (%)	94	9.0 ± 3.9^a^	5.3 ± 2.6
Age			
< 50	103	8.4 ± 3.8	5.7 ± 2.7
≥ 50	101	8.0 ± 3.5	5.2 ± 2.6
Department			
General surgery	87	8.5 ± 3.7	5.4 ± 2.6
Orthopedic surgery	82	8.0 ± 3.3	5.7 ± 2.6
Otorhinolaryngologic surgery	35	7.9 ± 4.3	5.1 ± 2.9
History of previous surgery			
YES	90	8.1 ± 4.0	5.0 ± 2.6
NO	114	8.3 ± 3.4	5.8 ± 2.6
ASA Class			
ASA I	103	8.0 ± 3.6	5.5 ± 2.6
ASA II	76	8.4 ± 3.9	5.1 ± 2.6
ASA III	25	8.1 ± 2.9	6.3 ± 2.8
Surgery			
Major	33	9.6 ± 3.8^b^	5.2 ± 2.5
Medium	47	8.7 ± 3.5	5.5 ± 2.8
Minor	124	7.6 ± 3.6	6.4 ± 2.8
Education level			
Primary Education	88	8.2 ± 3.8	5.4 ± 2.8
Secondary Education	71	8.0 ± 3.8	5.2 ± 2.6
Higher Education	45	8.6 ± 3.0	5.8 ± 2.4

^a^Independent-samples t test (t = 2.84, *P* < 0.01)

^b^Analysis of variance (F = 4.14, *P* < 0.05)

The female participants had significantly higher anxiety scores than the males (7.54 ± 3.27 vs 8.97 ± 3.93; *P* < 0.01). The patients undergoing major surgery had higher anxiety scores than those having medium-minor operations (F = 4.14, *P* < 0.05). There were no differences observed in the ratings of the APAIS anxiety score regarding age, department, ASA class, prior surgical procedures, or educational level. Patients with APAIS information sub-scale score ≥ 8, (score ranges: 2–4, 5–7, and 8–10) had a higher anxiety score (F = 17.86, *P* < 0.01).

### Reliability and validity of Chinese APAIS

The internal consistency of APAIS assessed using Cronbach’s alpha was 0.862 for the anxiety sub-scale and 0.830 for the information sub-scale. The construct validity was investigated using factor analysis. An exploratory factor analysis (EFA) with an oblique rotation of 99 subjects revealed two factors, which explained 76.45% of the total variances: anxiety (items 1, 2, 4 and 5) and the need for information (items 3 and 6). Communalities, factor loadings, eigenvalue, and the percent of variance in a two-factor solution are shown in Table 3. A confirmatory factor analysis (CFA) of 105 participants showed a two-factor model with an adequate model fit, and Goodness of fit index (GFI) and Root mean square error of approximation index (RMSEA) were 0.966 and 0.073, respectively. The indexes of model fitting are shown in Table 4. The criteria validity was evaluated by measuring the correlation between the APAIS anxiety score and the STAI-S scores. The mean scores of STAI-S and VAS-A were 38.7 ± 11.2 and 3.2 ± 2.5, respectively. APAIS strongly correlated with STAI-S (*r* = 0.717, *P* < 0.01) and VAS-A (*r* = 0.720, *P* < 0.01).

**Table 3:** Communalities, factor loadings, eigenvalue, percent of variance in a two-factor model

	Communalities	Factor Lodings		Eigenvalue	Percent of variance
		F1	F2		
A1	0.60	−0.70	0.33	3.59	46.53%
A2	0.69	−0.78	0.28		
A4	0.84	−0.89	0.19		
A5	0.79	−0.87	0.17		
A3	0.88	0.20	−0.92	1.00	29.92%
A6	0.79	0.29	−0.84

**Table 4:** Index of model fitting of a Two-Factor model

df	χ^2^	χ^2^ /df	*P* Value	NFI	CFI	GFI	RFI	RMR	RMSEA
7	10.86	1.551	0.145	0.965	0.987	0.966	0.925	0.049	0.073

### Coping style and preoperative anxiety

For the 101 inpatients who completed the MCMQ, the mean values of confrontation, avoidance, and resignation were 18.8 ± 3.4, 15.5 ± 3.2, and 7.5 ± 2.4, respectively. The correlations between the APAIS anxiety score, information desire score, and the MCMQ are shown in Table 5. At the APAIS anxiety sub-scale cut-off score of 11, 33.7% of the patients were classified as anxious. For the anxious inpatients, the t-test revealed the differences of each of the three factors: confrontation (t = 2.45, *P* = 0.016), avoidance (t = 0.32, *P* = 0.751), resignation (t = 1.38, *P* = 0.169), and the information desire score (t = 2.73, *P* = 0.007).

**Table 5:** The Pearson correlations of total anxiety, information desire and the coping styles

	Confrontation	Avoidance	Resignation	Information desire
Total anxiety	*r* = 0.33, *P* < 0.01	*r* = 0.07, *P* > 0.05	*r* = 0.22, *P* < 0.05	*r* = 0.40, *p* > 0.05
Information desire	*r* = 0.23, *P* < 0.05	*r* = −0.10, *P* > 0.05	*r* = 0.01, *P* > 0.05	1

## Discussion

The psychometric evaluation of the Chinese version of APAIS met the expected criteria. The scales showed a high reliability for both anxiety sub-scale and information sub-scale with Cronbach’s alpha 0.862 and 0.830 respectively. The criteria validity of Chinese version of APAIS was measured by the correlation with STAI-S, which is considered as gold standard for assessing state anxiety and the calibration of original version of APAIS, showed good efficiency [5, 17]. As in the original study two factors were extracted by EFA, the same results were also observed in our research. The Chinese APAIS differs from the French version with a three-factor structure and the Spanish version with one factor analyzed by CFA [9, 11]. We further verified the two-factor model for Chinese participants, and the results showed an adequate model fit. The CFA two-factor model coincided with EFA outcomes, which ensured stable construct validity for the new version of APAIS.

Unspecific anxiety questionnaires such as STAI-S were impractical and time-consuming. In our study, APAIS could be completed in 2 minutes with a high response rate and good acceptability in Chinese patients. In pilot testing, 30 participants were asked to rate the items of the scale using a dichotomous scale (clear or unclear) All patients reported clear and no misunderstanding. The possible reasons might be that, first the response format of APAIS was simple, sentence patterns of the six items were similar and colloquial. Second, as a condition-specific scale, APAIS describing surgery and anesthesia was pertinent and accessible in preoperative stage. Third, in the processes of translation/back translation, we solve most issues about words, sentence structure, semantics.

The APAIS contains two subscales: the anxiety scale has already been sufficiently studied, whereas there was relatively little research on the information scale that detects patients coping styles [8, 9]. Coping, defined as the cognition and behaviors to manage the internal and external demands in stressful situations, was a relatively stable characteristic [20, 21]. In our research, the mean information desire sub-scale score was 5.4 ± 2.6, lower than the sample in developed countries, which means more patients choose “blunting” coping style in dealing with surgery and anesthesia in China. It is possible that coping styles and subjective emotions are different across cultures [22]. However, patients with high information requirements or confrontation coping style still had higher anxiety levels [7, 9]. The explanation could be confrontation tended to focus on health-related threatening situations and paying close attention to details, which was an anxiety-provoking way to deal with uncontrollable stressors [23]. Furthermore, the monitoring style was an independent predictor of postoperative pain [24]. Avoidance and resignation were two additional types of coping strategy. Patients with resignation were focused on feelings of impotence and defeat [25]. Avoidance coping tended to avert stressful situations and avoid medical information. The relativities between these two coping styles and preoperative anxiety or information desire were not found in the study.

In addition, we found that the patients undergoing major surgery were more anxious, but the sample size of major surgery was small. Further qualified research is needed to confirm this result. One limitation of this study was that the research sample only included patients undergoing orthopedic, otolaryngologic, and general procedures. Although the range of surgery types was relatively narrow, in the original APAIS and other adaptions, there are was no interdependency between the anxiety levels and the types of procedures. The inpatients of the three departments were considered adequate to meet research needs.

## Conclusion

The Chinese version of APAIS is a valid and reliable instrument for assessing preoperative anxiety in pre-operative interviews. Using this instrument in Chinese patients is feasible and shows promising results.

## Data Availability

The dataset used during the current study is available from the corresponding author on reasonable request.

## Abbreviations

APAIS: The Amsterdam Preoperative Anxiety and Information Scale
CFI: Comparative fit index
CI: Confidence interval
EFA: Exploratory factor analysis
GFI: Goodness of fit index
ICC: Intraclass correlation coefficient
NFI: Normed fit index
RMSEA: Root mean square error of approximation index
STAI-S: State-trait anxiety inventory-state
VAS-A: Visual analogue scale-anxiety
